# Amyloid goiter: two cases and a review of the literature

**DOI:** 10.4103/0256-4947.51808

**Published:** 2009

**Authors:** Levent Yildiz, Mehmet Kefeli, Behiye Kose, Sancar Baris

**Affiliations:** From the Faculty of Medicine, Department of Pathology, Ondokuz Mayis University, Samsun, Turkey

## Abstract

Although involvement of the thyroid gland by amyloid is a relatively common phenomenon, clinically significant enlargement of the thyroid owing to amyloid deposition is an extremely rare occurrence. We describe two cases of amyloid goiter and review the relevant literature. The first case was systemic amyloidosis secondary to familial Mediterranean fever. The second case was a chronic renal failure patient who presented with an enlarged thyroid and upper airway obstructive symptoms. To date, true amyloid goiter secondary to amyloidosis associated with familial Mediterranean fever has only been reported in twelve patients.

Although involvement of the thyroid gland by amyloid is a relatively common phenomenon, clinically significant enlargement of the thyroid owing to amyloid deposition is an extremely rare occurrence. We describe two cases of amyloid goiter and review the relevant literature. The first case was systemic amyloidosis secondary to familial Mediterranean fever. The second case was a chronic renal failure patient who presented with an enlarged thyroid and upper airway obstructive symptoms. To date, true amyloid goiter secondary to amyloidosis associated with familial Mediterranean fever has only been reported in twelve patients.

Amyloid goiter can be defined as the presence of amyloid within the thyroid gland in such quantities as to produce a clinically apparent enlargement of the gland. This unusual entity was first described in 1858 by Beckman, and in 1904 by Eiselberg, who advanced the name “amyloid goiter”.^1^,^2^ Focal microscopic deposition of amyloid within the thyroid gland might be seen in systemic secondary amyloidosis, medullary thyroid carcinoma and less frequently, in primary amyloidosis.^1^,^3^ Clinically detectable thyroid enlargement from amyloid infiltration is rare, and most cases are not diagnosed prior to surgery. Amado et al reported 80 cases in a review of the world literature, most of which occurred in adults, although a few occurred in children.^4^,^5^ To date, true amyloid goiter secondary to amyloidosis associated with familial Mediterranean fever (FMF) has only been reported in twelve patients.^6^ The thyroid gland may be asymptomatically involved in most patients with systemic amyloidosis secondary to FMF. In this report, we describe two patients with amyloid goiter and we review the relevant literature.

## CASE 1

**Figure 1:**
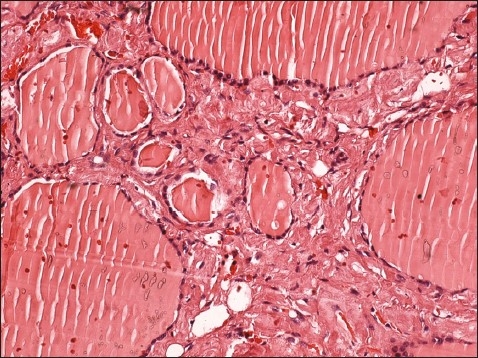
Diffuse eosinophilic amorphous material within interfollicular sites (hematoxylin-eosin, original magnification ×200).

**Figure 2:**
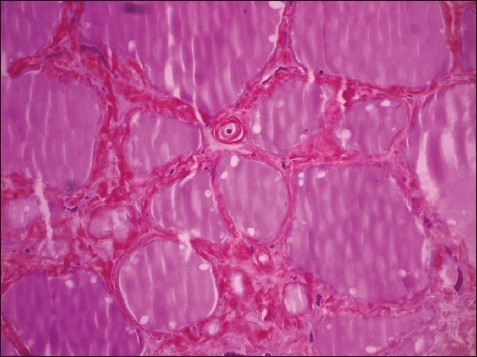
Methyl violet stain was metachromatically positive in the interfollicular sites (methyl violet, original magnification ×200).

A 16-year-old girl was diagnosed with FMF at the age of 10 years and was given colchicine. The patient was diagnosed as having secondary amyloidosis based on a renal biopsy performed to find out the cause of her clinical symptoms. On her last physical examination she had a bilateral, firm and diffusely enlarged thyroid gland. Her serum T3, T4, and TSH levels were all normal. A subtotal thyroidectomy was performed, with no complications. Grossly, the cut surface of the subtotal thyroidectomy material had a solid, white to pale-tan appearance and measured 8×6×3.5 cm. Microscopic examination by hematoxylin-eosin stained sections taken from both lobes of the thyroid revealed extensive infiltration of the parenchyma by an eosinophilic amorphous material consistent with amyloid substance (Figure 1). There was extensive fat cell metaplasia in the thyroid interstitium. The lining cells of the thyroid follicles were flattened and atrophic. There was no evidence of malignancy. Methyl violet stain was metachromatically positive in the involved tissue. This material stained intensely with Congo red and was apple green in color when examined microscopically under polarized light (Figure 2). Immunohistochemical staining patterns were consistent with amyloid AA (Clone mc1, Novocastra).

## CASE 2

**Figure 3:**
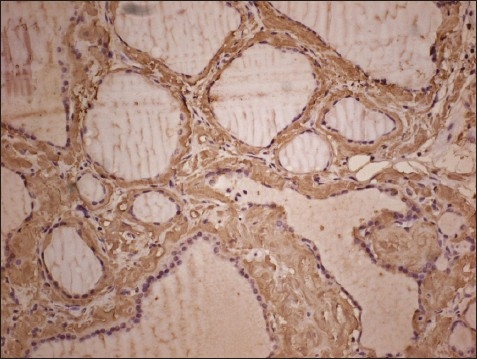
Immunohistochemically, eosinophilic material was positive with Amyloid AA (Avidin biotin, DAB Chromogen, original magnification ×200).

A 33-year-old male with secondary amyloidosis and chronic renal failure was admitted with rapidly growing goiter associated with hoarseness. Serum levels of the thyroid hormones and TSH were all normal. The patient underwent a subtotal thyroidectomy. The resected portion of the right lobe was enlarged and measured 10.5 cm in its greatest dimension. The resected portion of the left lobe was enlarged and measured 11 cm in greatest dimension. On gross examination of the thyroid gland, cut surfaces of both lobes of the thyroid had a largely solid, yellow-tan and irregular appearance. Both of the thyroid lobes were largely replaced by ill-defined solid, yellow-tan, fatty lesions. Microscopically, eosinophilic amorphous deposits were identified in interfollicular and perifollicular locations displacing and compressing the follicles. Areas of mature adipose tissue were seen intermixed with residual thyroid parenchyma and the eosinophilic amorphous deposits. Methyl violet stain was metachromatically positive in the involved tissue. These deposits stained intensely with Congo red and were apple green in color when examined microscopically under polarized light. Immunohistochemical evaluation demonstrated the presence of amyloid AA immunoreactivity (Clone mc1, Novocastra) (Figure 3).

## DISCUSSION

Amyloidosis results from the deposition of insoluble, fibrous amyloid proteins, nearly always in the extracellular spaces of organs and tissues. Named by Virchow in 1854 on the basis of the color after staining with iodine and sulfuric acid, all amyloid proteins share a unique fibrillar ultrastructure. Depending upon the biochemical nature of the amyloid precursor protein, amyloid fibrils can be deposited locally or systemically, involving virtually every organ system of the body. Amyloid fibril deposition may have no apparent clinical consequences or may lead to severe pathophysiologic changes. Regardless of etiology, the clinical diagnosis amyloidosis is usually not made until the disease is far advanced.^7^

Amyloidosis can involve the thyroid as part of a systemic disease or as a localized primary amyloid tumor. Amyloid goiter is extremely rare and presents more frequently as an autopsy finding than in surgical material. The thyroid gland may be asymptomatically involved by amyloid substance in nearly 30% to 80% of patients with primary or secondary amyloidosis.^1^,^8^ Amyloid substance might also be encountered in the thyroids of 50% to 80% of patients with medullary thyroid carcinoma.^6^ However, amyloid goiter, which is a symptomatic mass or clinically detectable thyroid enlargement due to amyloid deposition, is rare.^2^ The lesion may be unilateral or bilateral and is commonly associated with a foreign body type reaction. The amyloid deposits are often accompanied by mature adipose tissue.^9^ In the presented cases there was no foreign body type reaction, but in one patient extensive fat cell metaplasia in the thyroid interstitium was noted.

Amyloid goiter may be associated with either primary or secondary amyloidosis.^1^ In Turkey, FMF is the most common cause of secondary amyloidosis. The most common clinical manifestation of FMF-related amyloid is the development of the nephrotic syndrome and eventually uremia.^10^ However, the initial presence of a goiter caused by amyloid deposition is uncommon. Twelve cases of amyloid goiter complicating FMF were reported in the literature.^6^ In one of our patients amyloid goiter was also associated with FMF. Most commonly amyloid goiter presents as a rapidly growing neck mass causing compression symptoms including dyspnea, dysphagia and/or hoarseness.^11^ The enlargement of the gland is relatively rapid, occurring in weeks to several months. It usually presents as diffuse thyroid involvement, affecting both lobes, with a certain nodularity and associated firmness.^2^ In our patients, one had a rapidly enlarged thyroid associated with hoarseness. The other patient was diagnosed with FMF with secondary amyloidosis and on her last physical examination she had a bilateral, firm and diffusely enlarged thyroid gland. Altparmak et al have reported three patients with amyloid goiter in FMF, although none of their patients had rapidly growing thyroids.^6^

The diagnosis of amyloid goiter should be considered in any patient with systemic amyloidosis presenting with an enlarging diffuse goiter and euthyroid state. In patients with amyloid goiter, thyroid function tests are often non-specifically altered, and most patients are clinically euthyroid despite the diffuse involvement by the disease.^12^ However, there are rare reports of patients with hypothyroidism or hyperthyroidism.^13^,^14^ As previously stated, our patients had normal levels of T4, T3 and TSH.

The definitive diagnosis must be made by histologic evaluation of the resected thyroid gland, and although a fine needle aspirate may result in a definitive diagnosis, examination of resected tissue is usually necessary. Amyloid is usually present extracellularly as an amorphous, eosinophilic, proteinaceous substance in the light microscope. In cases of amyloid goiter, amyloid material is commonly seen infiltrating the parenchyma, distorting the normal tissue architecture. Other histologic features occurring in amyloid goiter include large foci of fatty metaplasia and rarely, squamous metaplasia as demonstrated by one of our patients as well. Histochemical stains aid in the confirmation of amyloid. These stains include Congo red, thioflavin T, and crystal violet stains. Congo red, the most frequently used technique, imparts a unique apple green birefringence when viewed under polarized light and is considered a pathognomonic feature of amyloid. Immunohistochemical techniques may help differentiating amyloid A from other types of amyloid.^2^,^15^

The presence of amyloid in the thyroid gland must raise the differential diagnosis of medullary carcinoma of the thyroid. In some cases amyloid may appear to almost completely obscure the underlying carcinoma. However, the lack of calcitonin reactive neoplastic cells and the presence of a background of C-cell hyperplasia can help to make this distinction. Amyloid deposition can also be seen in other conditions such as multiple myeloma, solitary plasmacytoma, infections, FMF, rheumatoid arthritis and a hyalinizing trabecular adenoma. However, these lesions each have a unique and different histologic appearance which renders discrimination easy.

The preoperative diagnosis of amyloid goiter should be suspected in patients with longstanding predisposing diseases such as FMF, hemodialysis, or with known amyloidosis who present with a rapidly growing diffuse goiter associated with euthyroid state. Histopathologic confirmation of the diagnosis is essential. We have described two patients with extensive involvement of the thyroid gland by amyloid substance, characteristic of amyloid goiter.
